# The Emerging Biomarkers in Chronic Obstructive Pulmonary Disease: A Narrative Review

**DOI:** 10.3390/diagnostics15101245

**Published:** 2025-05-14

**Authors:** Kaitlin M. Phillips, Philip F. Lavere, Nicola A. Hanania, Muhammad Adrish

**Affiliations:** Section of Pulmonary, Critical Care, and Sleep Medicine, Baylor College of Medicine, Houston, TX 77030, USA; kaitlin.phillips@bcm.edu (K.M.P.); philip.lavere@bcm.edu (P.F.L.); hanania@bcm.edu (N.A.H.)

**Keywords:** biomarkers, COPD endotypes, FENO, eosinophilic COPD, quantitative computerized tomography

## Abstract

The burden of chronic obstructive pulmonary disease (COPD) is increasing, especially for women in low-to-middle income countries. Biomarkers provide ever-increasing diagnostic precision for COPD and show promise for primary, secondary, and tertiary disease prevention. This review describes emerging applications for biomarkers in COPD, especially as they align with the Global Initiative for Chronic Obstructive Lung Disease (GOLD) emphasis on prevention, early diagnosis, and response to therapy. These biomarkers include blood eosinophils; IgE; C-reactive protein; fibrinogen; procalcitonin; interleukins 6, 8, and 33; tumor necrosis factor alpha; and soluble receptor for advanced glycated products (sRAGE). They have been used in various ways to identify COPD endotypes, predict exacerbations, predict mortality, and monitor the response to therapy. The fraction of exhaled nitric oxide (FENO) is increasingly studied in eosinophilic COPD endotypes and can be a diagnostic and predictive non-invasive biomarker. Imaging biomarkers, especially the quantitative computerized tomography (QCT) assessment of airway remolding, functional small airway disease, air trapping, lung function, and volume surrogates, all serve as non-invasive biomarkers for screening, early detection, and disease progression. Biomarkers facilitate all the phases of COPD care from detecting early airflow obstruction to predicting exacerbation and mortality. Biomarkers will be increasingly used as precise diagnostic tools to improve the COPD outcomes. The aim of this narrative review is to summarize the recent investigations in COPD biomarkers and their clinical applications.

## 1. Background

Biomarkers are widely utilized tools to inform clinical decision making. In recent years, the landscape of biomarkers in chronic obstructive pulmonary disease (COPD) has evolved, but identifying clinically relevant biomarkers has been challenging due to the heterogeneity of pathophysiologic airway and alveolar injuries, respiratory symptoms, and variable COPD progressions. Many COPD biomarkers are well-described inflammatory mediators of airway and parenchymal destruction [1]. Clinical COPD phenotypes are observable traits, while COPD endotypes are the biopathological mechanistic features of an individual’s disease [2]. While biomass burning smoke exposure is a significant risk factor for developing COPD, the majority of COPD biomarker research has been conducted on COPD cases related to tobacco smoking. Phenotyping COPD is straightforward, but identifying patient endotypes allows for more personalized care in the era of precision medicine. Clinical biomarkers are an important tool in describing a patient’s endotype in COPD. This review summarizes the recent developments in the COPD biomarkers of various media, including serum, sputum, exhaled, and radiographic features, and describes how these biomarkers can be utilized to define patient endotypes for precision medicine in clinical practice.

## 2. Overview of Serum Biomarkers in COPD

### 2.1. Blood Eosinophil Count

Eosinophils are a type of granulocyte that creates and releases pro-inflammatory mediators within the inflammation cascade. While they represent a small proportion of leukocytes in healthy patients, the amount of eosinopoiesis is increased in type 2 inflammatory states [3]. Elevated eosinophil levels (>300 cells·µL^−1^) have become a widely used indicator for type 2 inflammation, so their role as a practical and clinically useful biomarker in airway disease has exponentially grown in recent years [1]. Although elevated blood eosinophil levels are traditionally associated with asthma, patients with both asthma and COPD overlap (ACO), and some patients with COPD have elevated peripheral eosinophils [4]. Over the last few decades, an elevated blood eosinophil count has been identified as an endotype that is associated with the moderate and severe “frequent exacerbation” phenotype of COPD (requiring hospitalization) [4,5]. However, patients with elevated blood eosinophil levels have lower mortality rates both in hospitalized acute exacerbations of COPD and longitudinally over time, theoretically related to a better response to systemic corticosteroids [6,7].

An increased blood eosinophil count has also been associated with an accelerated decline in lung function. One study demonstrated that patients with elevated blood eosinophil levels demonstrated a faster decline in FEV1 by an average of 34 mL/year, regardless of their COPD status [8]. The large-scale Copenhagen General Population Study found that patients with chronic airway disease that were found to have an elevated blood eosinophil count and fractional exhaled nitric oxide (FeNO) demonstrated a more rapid decline in FEV1 [9]. However, some studies have shown that changes in serum eosinophils over time are not associated with changes in pulmonary function or health status [10] (Table 1).

The Global Initiative for Chronic Obstructive Lung Disease (GOLD) Report now recommends including blood eosinophil counts as part of the initial assessment of newly diagnosed COPD because blood eosinophil counts have proven useful pharmacodynamic biomarkers for COPD. Patients with elevated blood eosinophil levels (initially defined as at least 2% of blood eosinophils in the leukocyte differential) respond better to inhaled corticosteroid-containing regimens, specifically with reduced exacerbations, when compared to patients with normal blood eosinophil levels [11,12,13]. The FORWARD study also demonstrated that patients with blood eosinophil counts (defined as >279.8 in this study) who were treated with ICS in addition to LABA showed improved respiratory symptoms and pre-bronchodilator FEV1 when compared to those of similar patients using LABA alone [12]. Conversely, the FLAME study evaluated the role indacaterol–glycopyrronium versus salmeterol–fluticasone in reducing the risk of COPD exacerbations and found that the LABA-LAMA combination was more effective in preventing exacerbations in patients who had a COPD exacerbation within the last year, regardless of their blood eosinophil level (though patients with very high eosinophils as defined as >600 were excluded from analysis) [14]. This study further emphasized the role in LAMA therapy in COPD management regardless of blood eosinophil level. Later, the analysis of the ETHOS trial demonstrated that triple therapy with budesonide/glycopyrronium/formoterol fumarate dihydrate reduced the rates of moderate and severe exacerbations with blood eosinophil counts as low as ≥100 cells/mm^3^ [15]. Studies like these affirm the role of the blood eosinophil count as a useful clinical biomarker for predicting response to ICS.

As such, the GOLD Report recommends that patients with COPD with blood eosinophil counts >300 cells/uL are at higher risk for exacerbation and would likely benefit from ICS in their treatment plan [16]. Some studies discuss monitoring blood eosinophil counts as a measure of the ICS treatment response, though more investigation is necessary in this area. A recent European study demonstrated that initiation of ICS demonstrated a median reduction in blood eosinophils by 197 197 cells/μL [17]. These patients notably had an average of 1.71 exacerbations per year after starting ICS treatment. A proposed algorithm involves monitoring the blood eosinophils of people on inhaled corticosteroid treatment to determine if those patients benefit from continued ICS, although this approach is theoretical based on the available data and has not been validated. In this theorized approach, if the level of blood eosinophils rises after the initiation of ICS, then the ICS should be discontinued as this predicts someone who will not benefit from ICS and would needlessly be exposed to its side effects, such as dysphonia, topical candidiasis, skeletal effects, ocular effects, and pneumonia [10]. In fact, patients with COPD and low blood eosinophil counts (those less likely to benefit from ICS) that are receiving inhaled corticosteroids are associated with a higher risk of developing pneumonia [10].

While pharmacodynamic biomarkers have played a large role in the development of biologic targets, and thus treatments for asthma, the role of biologics in COPD is newly emerging, and currently targets molecular agents that promote eosinophil migration. IL-4 and IL-13 are inflammatory cytokines within the Th2 pathway that recruit eosinophils and contribute to goblet cell hyperplasia, mucous secretion, and airway remodeling in COPD [18]. Dupilumab, a human monoclonal antibody that blocks the IL-4 and IL-13 pathways of eosinophil recruitment, has shown a promising clinical response in patients with asthma and is now approved in the setting of COPD. The BOREAS trial, and subsequently the NOTUS trial recruited patients who were at high risk for COPD exacerbations; they had been on triple-inhaler therapy with LAMA, LABA, and ICS, demonstrated blood eosinophil counts >300, and had at least two exacerbations or one severe exacerbation within the last year [1,18]. In these studies, dupilumab was shown to decrease patients’ annual COPD exacerbations and to improve FEV1 and FVC. IL-5 is another important target for biologics in asthma management given their role in promoting eosinophilic inflammation. However, biologics that target the IL-5 pathways have not shown to reduce the symptoms or exacerbations of COPD, despite a measured decline in the blood eosinophil count [18]. Hence, the utilization of the blood eosinophil count as a biomarker has limitations.

### 2.2. Immunoglobulin E

The increased production of IL-4 and IL-13 in the Th2-inflammatory state also promotes the B cell production of immunoglobulin E (IgE) after allergen exposure. Viral infections also independently promote IgE release by driving dendritic cells to recruit Th2 cells that cause IgE release [19]. The role of IgE levels in COPD exacerbations is controversial. Previously, IgE elevation was demonstrated in asthma or asthma–COPD overlap, but only limited data highlight the role of IgE in isolated COPD. Recently, the investigators who designed the COSYCONET and WISDOM cohorts reviewed specific characteristics of patients with COPD in Europe as they relate to IgE levels. Higher rates of IgE elevation are seen in patients with both COPD and asthma, especially in current smokers [20]. However, a prospective observational study in Vietnam did not show clinically significant differences in IgE levels between patients with diagnosed COPD and control patients, implying that there may be a geographic role of IgE in COPD [21]. The individuals from the Copenhagen General Population Study cohort with COPD and elevated IgE demonstrated higher rates of severe exacerbations and all-cause mortality, independent of blood eosinophil count [22]. Interestingly, higher proportions of men with COPD had elevated IgE, and increased levels of total IgE have been correlated with higher risk of exacerbations in men than women, but patient age was not correlated with the levels of IgE [20]. In the COSYCONET trial, patients with elevated IgE levels did not show significantly different baseline FEV1 values, diffusion capacities, and residual volumes at baseline, but patients with levels more than >91.5 were at risk for future decline in FEV1 [20].

There is a promising role for IgE as a predictor of the future treatment response. In the BOREAS trial, patients with baseline IgE > 100 who were treated with dupilumab had fewer exacerbations and had a greater improvement in FEV1 than those treated with placebo in the 52-week treatment period [18]. The NOTUS trial subsequently demonstrated that dupilumab decreased IgE levels over the treatment period [1]. A further investigation is needed to validate the predictive, prognostic, and pharmacodynamic roles of IgE in COPD, especially to determine if omalizumab, a monoclonal antibody that binds IgE, could have a role in COPD management in the future.

### 2.3. C-Reactive Protein

C-reactive protein (CRP) is an acute phase reactant produced by the liver in response to IL-6 activity and plays a role in activation of the complement cascade, and thus pro-inflammatory cytokines to support innate immunity and phagocytosis during acute infection [23,24]. In both research and clinical practice, CRP is the most commonly measured inflammatory biomarker and often manifests as an early sign of chronic inflammation in patients with COPD [25]. Though multiple studies have described the utilization of elevated CRP in acute exacerbations of COPD, CRP is frequently elevated in both stable and acute exacerbations of COPD [26]. However, the supportive evidence for the role of CRP as a clinical biomarker is conflicting. In the ECLIPSE study, patients with COPD showed higher rates of CRP elevation when compared to non-smokers and smokers without COPD [27]. A meta-analysis has demonstrated that higher levels of CRP in COPD are associated with increased rates of exacerbation, hospitalization, and early mortality [25,27,28,29]. While the SUMMIT trial demonstrated positive association between elevated CRP and mortality, it did not demonstrate associations with FEV1 decline, exacerbations, nor hospitalizations [4].

Recent studies have demonstrated that CRP may be able to assist in managing the acute exacerbation of COPD as bacterial versus non-bacterial. In a recent meta-analysis, elevated CRP levels showed the strongest evidence of predicting bacterial COPD exacerbations (by positive sputum culture) when compared to multiple other biomarkers, such as procalcitonin, sputum IL-8, and sputum TNF-a [30]. Elevated CRP levels are more often correlated with bacterial infection in the lower airways compared to procalcitonin. A study in the United Kingdom has assessed the utility of CRP as predictive of who would benefit from antibiotics in COPD exacerbations. They demonstrated that using CRP levels to guide antibiotic therapy versus using sputum purulence showed no increase in 30-day adverse events or treatment failure [31].

### 2.4. Fibrinogen

Fibrinogen is a slow-reacting positive acute phase reactant influenced by increased IL-6 levels that directly determines the erythrocyte sedimentation rate (ESR). In a multitude of disease states, elevated fibrinogen levels have been traditionally associated with increased all-cause mortality. The SUMMIT trial confirmed this association with mortality in the setting of COPD specifically [4]. Fibrinogen has demonstrated both predictive and prognostic capabilities in COPD and has been approved by the United States Food & Drug Administration (FDA) to be utilized as a biomarker in clinical trials describing COPD mortality and exacerbations [32,33]. The ECLIPSE study showed that patients with COPD show higher rates of fibrinogen elevation when compared to those of non-smokers and smokers without COPD [27]. The serum fibrinogen levels are also higher in patients with both asthma and COPD [26]. The IMPACT trial demonstrates that patients with COPD and elevated fibrinogen levels demonstrated increased rates of moderate and severe exacerbations [34]. A recent meta-analysis also described that elevated levels of fibrinogen are associated with a decline in FEV1 [28]. However, the SUMMIT trial did not find significant association between elevated fibrinogen levels and FEV1 decline, exacerbations, or hospitalizations [4]. Fibrinogen is often elevated in exacerbations, but fibrinogen levels unfortunately have not demonstrated enough sensitivity nor specificity to be validated as a predictive biomarker in this setting. Fibrinogen elevation takes time, which is a limitation in its utility in the acute setting [33]. Like in the case of CRP, further research is necessary to define the association with future exacerbations.

### 2.5. Procalcitonin

Procalcitonin is also an acute phase reactant. Cytokines like IL-6, TNF-α, and IL-1b stimulate the synthesis of procalcitonin by thyroid C cells. Its primary utility as a biomarker has been predicting which patients with acute exacerbations of COPD would benefit from antibiotics, and the FDA has approved the use of procalcitonin for starting and stopping antibiotics for those with lower respiratory tract infections [25]. Like CRP, multiple studies have associated elevated procalcitonin levels with positive sputum cultures in bacterial acute exacerbations of COPD. A few small studies have evaluated procalcitonin-guided algorithms to reduce antibiotic exposure in COPD exacerbations. These studies demonstrated successful reduction in antibiotic exposure, but have used different cut-offs anywhere between 0.03 and 1.03 to determine who would benefit from antibiotics in the setting of acute COPD exacerbation [30,35]. One meta-analysis demonstrated that a cut-off point of 0.76 ng/mL demonstrated the best area under receiver operating characteristic curve (ROC), with sensitivity of 92.5% and specificity of 78.95% [30]. However, a French study of 300 patients admitted to the ICU with COPD exacerbations demonstrated that using a procalcitonin-guided strategy to guide antibiotic management failed to reduce antibiotic exposure [36]. Given the controversy in recent studies regarding using procalcitonin in COPD exacerbations, the GOLD Report recommends against solely using procalcitonin-based strategies to determine whether patients with COPD exacerbations would benefit from antibiotics [16].

### 2.6. IL-6 and IL-8

In COPD, airway exposure to noxious fumes begins inflammatory cascades that lead to an extensive environment of signaling molecules at the cellular level that eventually promotes cellular injury and apoptosis. Pro-inflammatory cytokines such as IL-6 and IL-8 are expressed at higher levels in injured airway tissue, and expression is further propagated by heat shock protein (HSP)-70 at the cellular level. IL-8 is implicated in Th1 inflammation, while IL-6 is implicated in Th2 inflammation, further describing the role of both Th1 and Th2 inflammation in COPD. Some studies on airway pathology have shown increased numbers of cells that are immunoreactive to IL-6 and IL-8 in COPD, and IL-6 and IL-8 have demonstrated potential as prognostic biomarkers in COPD [3].

Patients with COPD show higher rates of serum IL-6 elevation when compared to those of non-smokers and smokers without COPD per the ECLIPSE Study [27]. Increased serum IL-6 levels are associated with increased rates of future COPD exacerbations and hospitalizations, but not associated with mortality [28,37,38]. However, one study that utilized data from the ECLIPSE cohort showed that elevated serum IL-8 levels were associated with increased 3-year all-cause mortality in patients with COPD [28]. While one observational study detected higher levels of serum IL-8 in acute COPD exacerbations, the role of measuring serum IL-6 and IL-8 levels in acute exacerbations is unclear as previous studies have presented conflicting results [28,39]. These biomarkers are difficult to measure in clinical settings.

### 2.7. IL-33/ST-2

IL-33 belongs to the IL-1 superfamily of cytokines, which plays a role in innate and adaptive immunity [58]. Specifically, IL-33 is secreted by the epithelial cells and plays a role in remodeling and tissue repair, along with tissue homeostasis, which it does by repressing expression for some inflammatory genes. As an alarmin, IL-33 binds to the ST-2 (suppression of tumorigenicity 2) receptor and alerts the immune system of tissue damage and active Th2 response [59]. Neutrophils, natural killers, T cells, epithelial cells, and goblet cells also express ST-2 receptors. Increased ST-2 and IL-33 expressions have been reported in COPD and have been linked with higher risk of exacerbations [60]. The earlier data from phase 2 studies have demonstrated the safety of antibodies targeting IL-33 and ST-2, as well as improvement in lung function and exacerbation risks. A phase 2a study of anti-IL-33 agent Itepekimab showed improvement in prebronchodilator FEV1 and reduced exacerbation risk in patients with COPD [61]. A phase 2a study of anti-ST-2 Astegolimab in patients with COPD did not improve the exacerbation rates, but showed improvements in FEV1, the symptom score, and the eosinophil count [62]. Further data from several ongoing phase 3 studies are expected to shed more light on the safety and efficacy of these agents [59].

### 2.8. Soluble Receptor for Advanced Glycation End Products

The soluble Receptor for Advanced Glycation End products (sRAGE) is a cleaved version of a transmembrane receptor categorized as an immunoglobulin gene [40]. Mediators in the inflammatory cascade create RAGE ligands that bind to the RAGE receptor. When the RAGE receptor is bound, it promotes the further transcription of inflammatory cytokines, creating an amplification cycle for RAGE binding, and thus the generation of an inflammatory cascade [2]. Decreased levels of sRAGE have been found in multiple chronic diseases, including chronic airway disease (COPD and asthma) [4]. However, sRAGE is often elevated in acute respiratory disease, including infection-driven acute respiratory distress syndrome (ARDS) and isolated RSV infection (in experimental models at least) [40]. The levels of sRAGE have been found to be lower in smokers without COPD than non-smoking populations, which suggests a role of smoking in the modulation of the genetic expression of sRAGE [2]. Although, a study in Mexico demonstrated that the sRAGE levels are decreased in patients with COPD due to both tobacco smoking and biomass burning smoke exposure when compared to those of control groups exposed to these known substances [63]. A meta-analysis by Pratte et al. summarized the findings of sRAGE clinical associations in COPD from four large assays: COPDGene, SPIROMICS, ECLIPSE, and Pittsburgh COPD SCOOR. Reduced levels of serum sRAGE were found to be associated with severe baseline airflow obstruction by FEV1 and emphysema on imaging at the time of investigation [40]. In fact, the levels of sRAGE closely relate to the GOLD classification of COPD severity [2]. However, these cohorts demonstrated inconsistent findings when attempting to associate a reduction in sRAGE and the progression of obstruction and/or emphysema [40]. The SUMMIT trial also did not find a significant association between the levels of sRAGE and FEV1 decline, exacerbations, hospitalizations, nor mortality [4]. The relationship between the sRAGE levels and COPD suggests that COPD may be affected by genetic contributions, an important subject that warrants further investigation.

### 2.9. Club Cell Secretory Protein

Club cell secretory protein (CC16) is made by pulmonary tissue and secreted by club cells during a significant lung injury to provide immunosuppressant and anti-inflammatory properties [2,41]. As the number of functional club cells declines during COPD disease development, less CC16 is secreted. CC16 shows promise as a novel biomarker in COPD. Patients with COPD are found to have lower circulating levels of serum CC16 (which is been associated with the bronchoalveolar lavage levels), particularly in male patients with advanced age [42]. Lower levels of CC16 are associated with a more rapid progressive decline in FEV1 (≥40 mL/yr) [2,42]. Changes in CC16 levels are not, however, associated with the progression of emphysema by imaging nor mortality in the ECLIPSE cohort. Similarly, its association with COPD exacerbations and hospitalizations has not been well studied. The role of CC16 in predicting other factors of disease progression aside from evidence of worsening airflow obstruction warrants further investigation.

### 2.10. Surfactant Protein-D

Surfactant protein D (SP-D) is another novel biomarker produced by type II pneumocytes that plays a role in the lungs’ innate immunity [2,43]. The serum levels of SP-D increase as a result of COPD-associated lung injuries and are found at higher levels in patients with COPD when compared to those of control subjects [2,41]. The SP-D levels are elevated in acute exacerbations of COPD, and stable patients with COPD with elevated serum SP-D have shown an increased risk of exacerbation within the following year [43]. The ECLIPSE study also demonstrated associations with elevated SP-D levels and 3-year mortality. Several studies have evaluated the association between SP-D levels and changes in spirometry, but with conflicting results. Its utility as a useful and specific predictive biomarker in COPD is limited in that the levels are associated with a variety of different factors, including age, body mass index, and the male sex. THe levels are also increased in multiple different pulmonary disease states [43].

## 3. Overview of Lung Samples in COPD

### 3.1. Sputum Biomarkers

**Figure 1:**
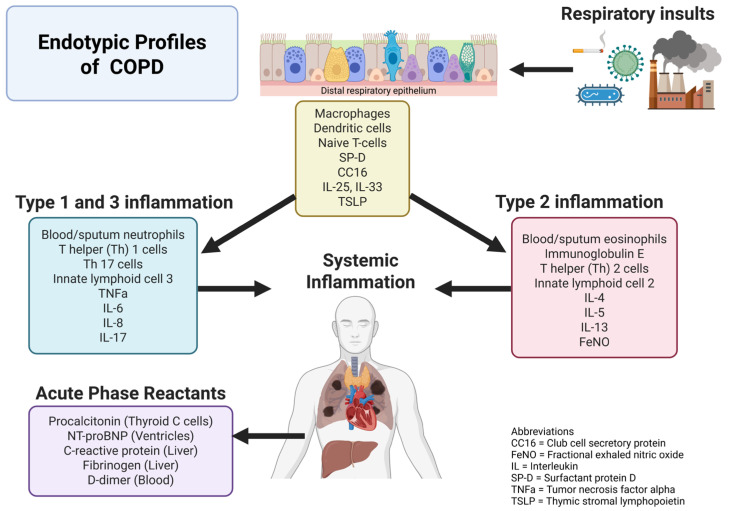
Endotypic profile of COPD (created with www.biorender.com).

Identifying sputum biomarkers in COPD is a growing area of interest. The roles of IL-6 and IL-8 in COPD pathogenesis have been described previously, and some studies have shown increased concentrations of sputum IL-6 and IL-8 in acute COPD exacerbations and severe chronic COPD when compared to those in stable or less-severe COPD, respectively [25]. Sputum IL-8 has demonstrated a potential role of differentiating bacterial versus non-bacterial exacerbations of COPD. Several small studies have assessed the sputum IL-8 levels and their association with bacterial exacerbations of COPD, but the results of these studies have been variable [30]. Tumor necrosis factor alpha (TNF-α) is a cytokine that contributes to propagation of the Th1 inflammatory response (Figure 1). The expression of TNF-α is significantly increased by airway tissue experiencing tissue and cellular injuries in COPD. Higher numbers of TNF-α immunoreactive cells have also been found in the airway tissue of patients with COPD [3]. Elevated levels of sputum TNF-α are seen in cases of severe COPD and acute exacerbations of COPD when compared to those in stable and less-severe COPD, respectively [25]. Multiple studies have evaluated the association between increased sputum TNF-α and the evidence of bacterial infection (highest in Pseudomonas aeruginosa infections), though all these studies were completed in an outpatient setting [30]. Despite the promising role of sputum measurements, the serum measurements of TNF-α demonstrate no statistically significant associations with mortality, exacerbations, or hospitalizations [28].

Many other sputum biomarkers have been associated with COPD, but the data regarding their diagnostic, predictive, prognostic, and therapeutic utility are limited. Sputum studies have shown that a subset of patients with COPD have eosinophil counts of >3%. Similarly, elevated eosinophil counts have also been noted in airway biopsy studies on patients with COPD exacerbation. Therefore, eosinophilic inflammation has emerged as a treatable trait in COPD due to the large number of targeted therapies being studied. Some studies have indicated that blood eosinophils correlate with sputum eosinophils to a lesser degree in COPD compared to patients with asthma. Elevated sputum eosinophils were associated with increased risks of exacerbation and lung function decline in the SPIROMICS cohort [64]. A small Colombian study reviewed the sputum biomarkers of women with COPD secondary to tobacco or wood burning smoke and demonstrated that the women with COPD demonstrate higher sputum levels of IL-8, metalloproteinase 9 (MMP-9), and chemokine ligand 5 (CCL5) than the control groups [65]. The investigators also reported higher levels of sputum CCL5 in COPD from tobacco smoking than disease secondary to wood burning, implicating a potential role for this biomarker to delineate between these two disease drivers, though more investigations are warranted. This study assessed the variation in sputum vascular endothelial growth factor (VEGF) levels in COPD and control patients, but did not detect a significant difference. However, a small Japanese study compared the VEGF levels in control patients and those with emphysema and chronic bronchitis and demonstrated that lower VEGF levels were associated with a decline in airflow obstruction in emphysema, and higher VEGF levels were associated with worsening obstruction in chronic bronchitis [66]. Further research is needed to evaluate the utility of these biomarkers in clinical practice.

### 3.2. Invasive Biomarkers

Many studies have also begun to explore the utility of invasive pulmonary samples, such as bronchoalveolar lavage (BAL) and lung biopsy, in identifying the clinically relevant biomarkers in COPD. Most studies that have analyzed BAL samples from patients with COPD have found higher levels of various inflammatory proteins when compared to those of healthy patients. One study evaluated the extracellular vesicle-associated protein and microRNA profiles in BAL samples from patients with COPD and found higher expression levels of 284 proteins, many of which were found to be involved in inflammatory mechanisms [67]. Few studies have assessed the clinical utility of this biomarker. BAL glutathione levels have been found to be reduced in acute exacerbations of COPD when compared to those of patients with COPD [25]. In a bronchoscopy sub-study of the SPIROMICS cohort, the investigators aimed to assess a BAL protein signature associated with FEV1 decline in COPD. They did not find a particular protein signature in the BAL protein analysis of this 25-patient cohort that predicted FEV1 decline given that their chosen assay had limited utility in studying BAL samples [68]. Unfortunately, many of the assays used to detect serum biomarkers have not been validated for BAL and lung biopsy samples, which has created barriers to research progress in this area. The role of lung biopsy in identifying the clinically relevant biomarkers in COPD has had limited investigation, and further research in this area is warranted.

### 3.3. FENO in COPD

Fractional exhaled nitric oxide (FENO) is a biomarker of Th-2 airway inflammation and has been extensively studied in asthma [44]. The emerging data show promise using FENO as a biomarker to endotype COPD. Two large clinical trials recently evaluated dupilumab for patients with eosinophilic-type COPD (defined by blood eosinophil counts >300 cells/uL). Interestingly, although >50% of the patients in both the trials had FENO < 20 ppb, the averaged baseline FENO for all the patients was 24 ppb, suggesting heterogenous endotypes of eosinophilic COPD with a range of FENO values. When the patients were stratified by FENO > 20, the NOTUS trial showed FEV1 improvements on dupilumab at 12 weeks, but not 52 weeks. Throughout the trial, FENO was reduced by roughly 10 ppb during therapy. These results are contrasted in the BOREAS trial, which when stratified by FENO > 20, the patients on dupilumab had sustained improvements in FEV1 and reduced moderate-to-severe COPD exacerbations at 52 weeks. Similar to NOTUS, FENO was roughly reduced by 10 ppb during treatment [1,18].

In addition to pharmacodynamic and monitoring applications, FENO can be used as a diagnostic, prognostic, and predictive biomarker in COPD. FENO may distinguish asthma from COPD or characterize asthma–COPD overlap (ACO) [45,46,47,48]. Non-smokers or former smokers with COPD have a lower FENO than those with asthma [49]. A 1-year prospective biomarker study found that measuring FENO combined with blood eosinophil counts improved the diagnostic accuracy for severe COPD exacerbations compared to that of each biomarker alone [50]. Other research suggests FENO does not improve the diagnostic utility of exacerbation, but it does distinguish COPD from ACO [51]. Persistent FENO ≥ 20 ppb in patients with stable COPD is associated with an increased risk of future exacerbations [52]. One study shows patients with COPD and elevated FENO levels experienced greater symptom control on ICS + LABA; however, even so, an older study suggests ICS has no effect on COPD lung function [53,54]. Patients with severe COPD who no longer smoke displayed concordant reductions in FENO alongside systemic markers of inflammation (CRP, IL-6, and IL-8), while receiving ICS [55]. A study investigated the effects of bronchodilation on the measurement of FENO and showed an increase in measured value after inhaled bronchodilator therapy [56]. In a small cohort of patients with stable COPD caused by either tobacco smoke or biomass burning smoke exposure, the FENO level was equally elevated compared to that of healthy controls [57].

### 3.4. Biomarkers of Exhaled Breath Condensate

Exhaled breath condensate (EBC) is a non-invasive method of assessing airway inflammation and oxidative stress in the airways, with potential implications in COPD diagnosis, phenotyping, and disease monitoring [69]. Several EBC biomarkers have been studied, including, but not limited to, leukotriene B4 (LTB4); prostaglandin E2 (PGE 2); 8-isoprostanein; nitric oxide (NO) metabolite; hydrogen peroxide (H_2_O_2_); lactate; formate; acetate; butyrate; malondialdehyde (MDA); matrix metalloproteinase-12 (MMP-12); neutrophil elastase (NE); and the tissue inhibitor of metalloproteinase-4 (TIMP-4); and cytokines such as IL-6, IL-10, and TNF-α [69,70,71].

The EBC profiles have shown the potential to detect subclinical COPD. In a study of 300 community participants, EBC, MDA, and lactate were able to discriminate even early-stage COPD in previously undiagnosed patients [71]. Additionally, the analysis of EBC profiles has been shown to differentiate between asthma and COPD. A study showed increases in ethanol and methanol levels and a significantly lower level of formate in the EBC of patients with COPD compared to patients with asthma [72]. Similarly, a decrease in methanol level along with improvement in walk distance and dyspnea was seen in the EBC of patients with COPD undergoing pulmonary rehabilitation [73].

Exhaled breath biomarkers have also been linked with COPD disease severity and exacerbation. A study comparing 161 patients with stable COPD and 112 controls showed that the EBC pH was lower in the patients with COPD [74]. Hydrogen peroxide, which is a direct marker of air-space oxidative burden, was noted to be elevated in the EBC of smokers and COPD subjects compared to that of non-smokers [75]. Another study evaluating neutrophil chemotactic activity and a neutrophil chemoattractant in EBC demonstrated higher levels in outpatients with COPD exacerbation compared to those of stable patients with COPD [76]. EBC can also identify bacterial nucleic acid in patients with COPD with exacerbations [77]. A prospective study of 68 patients evaluated their exhaled breath profiles before, during, and after COPD exacerbation and demonstrated classification accuracies of 71% for baseline vs. exacerbation and of 78% for exacerbation vs. recovery [78]. Elevated levels of H_2_O_2_ and 8-isoprostanein have also been shown to be linked with dyspnea sensation in COPD [79].

The study of EBC biomarkers is an evolving field, but challenges remain with regard to identifying reliable and clinically useful exhaled breath biomarkers that are accurate and effective for their intended use. Additional limitations include the lack of standardized interpretation for these biomarkers, as well as the need for a well-equipped laboratory to ensure an accurate performance [80].

## 4. Overview of Imaging Biomarkers for COPD

Numerous thoracic computerized tomography (CT) features are emerging biomarkers for COPD. Quantitative CT (QCT) imaging reliably characterizes the changes in obstructive lung disease, including air trapping, hyperinflation, mucus plugging, airway remodeling, and emphysema severity, among others. Advanced QCT techniques elucidate more nuanced and dynamic disease classifications within COPD. For example, parametric response mapping (PRM) identifies the heterogenous air trapping regions between emphysema and functional small airway disease (fSAD) and strongly correlates with all the stages of COPD severity [81,82,83]. Research on QCT for COPD has proliferated due to the availability of imaging datasets from several large cohort studies and provides new tools for COPD screening, early diagnosis, monitoring disease progression, predicting lung function decline, and prognosticating exacerbations and mortality (Table 2).

### 4.1. Changes in Airway Anatomy

In patients with COPD, mucus plugs obstructing medium-to-large airways are associated with higher mortality [84]. Silent mucus plugs are very common in patients who smoke tobacco [85]. In patients without cough or phlegm, silent mucus plugs are associated with poor disease control and a worse functional status [86]. Mucus plugs are linked to airflow limitation [87,88]. Airway wall thickness was associated with impaired quality of life, but not arterial oxygen tension [89,90]. In addition, airway wall thickness was associated with increased mortality as emphysema progressed [91]. Pathological airway remodeling with diminished airway branching complexity was prognostic of respiratory morbidity and lung function [92]. Ex-smokers were found to have thinner airway walls, which was linked to higher total (presumably recovered) airway counts [93]. Decreasing total airway counts are related to lung function decline, serving as an early imaging biomarker for COPD progression [94]. Changes in the airway-surface-area-to-volume ratio are associated with respiratory morbidity, COPD progression, and mortality [95]. A cohort of 400 patients with COPD with increasing Pi10 values (normalized index of airway thickness) could predict exacerbation risk, lung function decline, and mortality [96]. Current or former smokers with normal spirometry, but airway wall thickening on QTC, have exacerbations and functional limitations [97].

### 4.2. Developing and Progressing COPD: Emphysema and Lung Function

In one cohort of patients with clinical symptoms of COPD and significant emphysema, spirometry was normal 10% of the time [98]. Visual emphysema in smokers at GOLD stage 0 predicted structural physiological disease progression [99]. QCT determines the percentage of low-level attenuation areas on expiration, indicating regions of air trapping, which are strongly associated with airflow obstruction on spirometry [100]. QCT on emphysema is correlated with the diffusion capacity of the lungs for carbon dioxide [101]. In a 5-year cohort of patients actively smoking tobacco, most emphysema progression was not accounted for by the FEV1 trends, but rather the QCT measures of air trapping [102]. Pan lobular emphysema is associated with greater airflow obstruction, increased respiratory symptoms, and systemic inflammation [103]. Severe centrilobular emphysema was linked to worsening diffusion capacity and higher mortality in severe GOLD stage [104]. Higher grades of emphysema on visual grading and artificial intelligence deep learning were associated with disease progression across all the GOLD stages [105,106]. Emphysema progression on QCT is associated with increased all-cause respiratory mortality [107].

Functional small airway disease (fSMAD) is associated with FEV1 decline in mild-to-moderate COPD [112]. The spatial analysis of coalescing fSMAD pockets predicted lung function decline and identified regions of emphysema onset [108]. The regions of fSAD to emphysema transition may serve as an imaging biomarker that identifies otherwise healthy patients at risk for developing COPD [113]. Decline in lung density was associated with the serum biomarkers SP-D and sRAGE [114]. Centrilobular emphysema is associated with sRAGE [115]. Declines in quantified total lung capacity are associated with an FEV1 increase in PRISm, but FEV1 decreases through GOLD stages 3–4 [116]. Over 10 years, patients actively smoking with emphysema had the greatest decrease in lung density [117]. The QCT markers linked with spirometry and the development of COPD include FEV1 and airway wall thickness, FEV1/FVC and emphysema, air trapping and residual volume, functional small airway disease and FEV1, and lung volumes with COPD progression [112,118,119].

Disease progression modeling characterizes developing COPD subtypes. One study found a “tissue to airway” pattern of fSAD and emphysema before large airway changes (airway wall thickness and airway wall area), whereas the “airway to tissue” pattern was reversed. Such QTC information applied across multiple biomarker domains, and indeed 30% of healthy smokers, showed features of both COPD pathologic patterns [120]. Two indices of CT-measured lung volume in patients smoking tobacco were successfully descriptive of emphysematous pathology (high TLC) versus airway disease/airflow obstruction (high FRC/TLC) [121].

### 4.3. Predicting Exacerbations by Imaging

The QCT markers of lung tissue texture and airway structure were predictive of exacerbations and performed better than BODE or exacerbation history [122,123]. Airway wall area and wall thickness were associated with the frequent COPD exacerbation phenotype in patients with stable disease [92]. Even when QCT was obtained for non-pulmonary reasons, the incidental finding of emphysema and airway wall thickness predicted future severe COPD exacerbations [124].

### 4.4. Pulmonary Vasculature and Other Imaging Markers

Some patients with mild COPD were found to have reduced pulmonary microvascular blood flow in non-emphysematous lung regions, a potentially distinct process, or in the “airway to tissue” subtype [125]. Pulmonary artery strain is reduced in COPD [126]. An increased residual volume in patients with COPD was linked to a larger pulmonary artery area [127]. Pulmonary artery pruning was associated with progressing emphysema, rapid lung function decline, increased residual volume, exercise capacity, and COPD mortality [109,110]. The total cross-sectional area of sub-segmental pulmonary artery vessels was correlated with the degree of emphysema [128]. Enlarging pulmonary arteries in patients with COPD were associated with lower exercise tolerance and increased mortality [111]. Hyperinflation is associated with coronary artery disease in patients who smoke [129].

### 4.5. Identifying Undiagnosed COPD by Imaging

COPD is highly prevalent in lung cancer screening populations [130]. Almost 2/3 of patients in a lung cancer screening cohort were found to have undiagnosed COPD [131]. In another large cohort of patients undergoing general lung health screening, almost 20% were found to have undiagnosed COPD [132]. One study found low-dose expiratory CT scans for male smokers undergoing lung cancer screening had 63% sensitivity and 88% specificity for diagnosing COPD [133].

## 5. Clinical Utility of COPD Biomarkers

**Figure 2:**
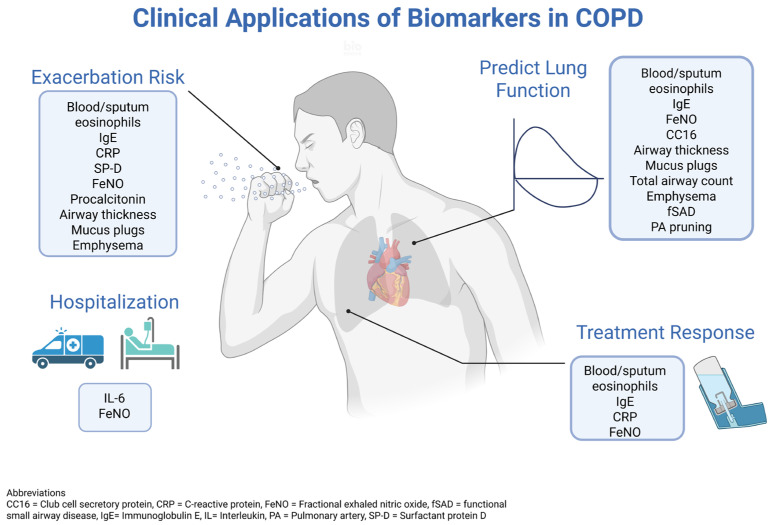
Clinical applications of biomarkers in COPD (created with www.biorender.com).

Physiological biomarkers have varying degrees of utility in the clinical evaluation and management of COPD. Blood eosinophil count is a crucial biomarker for identifying patients with type 2 inflammation who are more likely to benefit from inhaled corticosteroids, as well as biologics like dupilumab, and it also carries prognostic information concerning exacerbation frequency and lung function decline [1,18] (Figure 2). As such, the GOLD Report guidelines now recommend the measurement of blood eosinophil as a component of routine COPD evaluation [16]. IgE is emerging as an important prognostic biomarker and one that may also predict the response to dupilumab [18]. The systemic inflammatory markers CRP and fibrinogen offer predictive and prognostic values for exacerbations and mortality, with CRP also showing promise in guiding antibiotic use in the management of exacerbations. The role of procalcitonin in guiding antibiotic therapy in COPD exacerbations remains debated, but it remains a sound strategy to de-escalate antibiotics based on decreasing procalcitonin levels. The cytokines IL-6 and IL-8 have demonstrated prognostic associations with exacerbations, hospitalizations, and mortality, though their use in acute settings is less clear. Novel biomarkers, such as IL-33/ST-2, sRAGE, CC16, and SP-D, provide potential prognostic insights into exacerbation risk, disease severity, lung function decline, and mortality, but require further investigation of how this insight tailors management. Sputum biomarkers are being investigated for their ability to reflect airway inflammation and predict exacerbations, while invasive biomarkers from bronchoalveolar lavage and lung biopsies require more exploration. FeNO is proving useful in distinguishing COPD from asthma/ACO, predicting the exacerbation risk, and potentially monitoring and predicting the response to therapies targeting type 2 inflammation [44,45,46,47,48]. Exhaled breath condensate analysis offers a non-invasive approach to assess airway inflammation and oxidative stress, though the identification of reliable clinical biomarkers in this domain remains a challenge.

Currently, there are only a few clinical applications for imaging biomarkers in COPD. The guidelines highlight QCT for planning endobronchial valve placement or lung reduction surgery [16]. Specifically for bronchial valves, QCT identifies the emphysema lobe level, emphysema distribution, adjacent lobar volume, and fissure integrity, which are critical for proper patient and valve site selection [134,135,136]. Another clinical application for imaging biomarkers uses annual low-dose CT (LDCT) scans for lung cancer screening. One study reported 63% sensitivity and 88% specificity of LDCT for detecting COPD [133]. A very high prevalence of COPD has been identified in other patient cohorts undergoing annual LDCT, highlighting the clinical utility of LDCT for COPD diagnosis and population-level case findings [131,132,137].

In a few short years, we anticipate other emerging QCT techniques to enter clinical medicine. Emphysema percent quantification (somewhat analogous to the left ventricular ejection fraction) identifies the percent of low-level attenuation areas in pulmonary parenchyma, which are associated with COPD symptoms, airflow obstruction, disease progression, and mortality [100]. The systematic grading of airway mucus plug burden or small airway counts should become easier with artificially intelligent automated radiology software, and these findings have been associated with airflow obstruction, symptoms, and exacerbations [138]. Future challenges remain, such as defining optimal imaging protocols considering age, sex, and race, where meaningful clinical differences exist; proper artificial intelligence training on large imaging datasets; and further technological advancements in scanning resolution and software processing power.

Despite the growing interest in COPD biomarkers, several challenges hinder their widespread clinical application and incorporation into practice guidelines. Conflicting evidence across studies for these biomarkers creates uncertainty regarding their reliability and utility. A significant limitation is the lack of validation in large, prospective studies to confirm the clinical significance of these biomarkers and to establish reliable cut-off values for clinical decision making. Furthermore, the heterogeneity of COPD contributes to inconsistencies in the research findings. COPD is a complex disease that involves multiple inflammatory pathways and clinical phenotypes, so relying on individual biomarkers may not appropriately capture the multifaceted nature of the disease. Many of these biomarkers are altered in a multitude of other disease states, and there are a lack of validated data to support the specificity in utilizing these biomarkers in the clinical setting of COPD management. Lastly, while the emerging targeted therapies have highlighted the need for effective biomarkers, the translation of the research findings into routine clinical practice often faces hurdles related to standardization and cost-effectiveness. Overall, further research will be essential to validate the clinical utility of COPD biomarkers and to develop biomarker-driven strategies for precision COPD care.

## 6. Differential Biomarkers Between Tobacco Smoke and Biomass Burning Smoke Exposure

It is estimated that almost half of COPD worldwide is due to non-tobacco exposure [139]. Although the majority of COPD biomarker work has been conducted on patients exposed to tobacco smoke, (TS-COPD) there are emerging biomarker data for COPD related to biomass burning smoke exposure (BS-COPD). A cohort of over 700 patients from Morrocco who self-reported cumulative biomass burning smoke exposure (wood heating for men and cooking biomass for women) revealed increased odds of COPD with higher biomass burning smoke exposure indices [140]. The natural history of BS-COPD is different from that of tobacco smoking, with less parenchymal destruction, more chronic bronchitis, and the slower decline of lung function [141,142].

One small study found greater bronchial hyperresponsiveness in BS-COPD compared to TS-COPD [143]. Another study directly compared the cytokine profiles between 29 women with TS-COPD and 31 women with BS-COPD and found the level of chemokine ligand 5 was higher in the TS-COPD group [65]. Additional research has uncovered distinct features of BS-COPD compared to those of healthy controls; one cross sectional study of 180 women in rural Peru found that kitchen concentrations of black carbon were associated with increased blood levels of the pro-inflammatory cytokine TNF-alpha [144]. Another cohort of 140 women from East India who cook exclusively with biomass burning smoke exposure had elevated serum levels of TNF-alpha and neutrophils compared to those of women who cook with cleaner petroleum gases, and this finding was replicated in a larger cohort study involving over 1000 participants [145]. Conversely, a smaller case–control study from South India found that serum TNF-alpha levels were much higher in 40 patients with tobacco smoke COPD versus those of 40 patients with BS-COPD [146]. What is clear, however, is that more research is needed to understand the major BS-COPD phenotype.

## 7. Conclusions

Biomarkers have significant potential for improving the diagnosis, prognosis, and management of COPD. Serum, sputum, imaging, and exhaled biomarkers are becoming increasingly important tools in not only predicting COPD outcomes, but predicting the treatment response to evolving therapies in COPD. The integration of these biomarkers into clinical assessments promotes personalized treatment strategies, enabling more targeted interventions and better patient outcomes. However, important unmet needs remain, including the stability of biomarkers over time, the predictive ability of composite biomarkers, and the lack of well-defined type 1 biomarkers. Importantly, additional research that would clinically validate these various biomarkers longitudinally is warranted. An ongoing investigation of novel biomarkers, including other known inflammatory mediators and genetic markers, may also uncover new therapeutic targets, improve prognostic guidance, and further enhance the understanding of COPD pathogenesis. There has been immense development in investigating the role of biomarkers in COPD, but further research is needed to continue to progress COPD care and improve the quality of life for affected patients.

## Figures and Tables

**Table 1:** Association of serum/blood and exhaled breath biomarkers with different COPD outcomes.

Serum/Blood Biomarkers	FEV1 Decline	Exacerbations	Emphysema on Imaging	Hospitalization	Mortality	Predicting Response to a Treatment	References
**Eos**	↑	↑				↑ *	[3,4,5,6,7,8,9,10,11,12,13,14,15,16,17,18]
**IgE**	↑	↑				↑ **	[19,20,21,22]
**CRP**	↔	↑		C	C	↑ ***	[23,24,25,26,27,28,29,30,31]
**Fibrinogen**	C	C		↔	↑		[4,26,27,32,33,34]
**PCT**		↑	↔			C ***	[16,25,30,35,36]
**IL-6**				↑	↔		[3,27,28,37,38,39]
**IL-8**		C			↑		[3,28,39]
**sRAGE**	↔ ****	↔	↓	↔	↔		[2,40]
**CC16**	↓				↔		[2,41,42]
**SP-D**	C	↑			↑		[2,43]
**Exhaled Breath Biomarker**							
**FeNO**	↑	↑		↑	↑	↑ *	[1,18,44,45,46,47,48,49,50,51,52,53,54,55,56,57]

↑ = positive clinical association; ↓ = inverse clinical association; ↔ = no association identified; C = conflicting evidence; Eos = blood eosinophil count, IgE = immunoglobulin E, CRP = C-reactive protein, FeNO = fractional exhaled nitric oxide, PCT = procalcitonin, IL-6 = Interleukin-6; IL-8 = Interleukin-8; sRAGE = soluble receptor for advanced glycation end products, CC16 = club cell secretory protein, SP-D = surfactant protein-D; * inhaled corticosteroids, dupilumab; ** dupilumab; *** antibiotics in acute exacerbations; **** associated with baseline lower FEV1, but not decline in FEV1.

**Table 2:** Association of imaging biomarkers with different COPD outcomes.

Imaging Biomarker	Lung Function Decline	Risk for Exacerbation	Functional Status Decline	Mortality	References
Mucus Plugs	↑	↑	↑	↑	[84,85,86,87,88]
Airway Wall Thickness	↑	↑	↑	↑	[89,90,91,92,93,94,95,96,97]
Total Airway Counts	↑				[94]
Emphysema	↑	↑		↑	[98,99,100,101,102,103,104,105,106,107]
fSAD	↑				[108]
Pulmonary Artery Pruning	↑		↑	↑	[109,110]
Enlarging Pulmonary Arteries			↑	↑	[111]

↑ = positive clinical association; fSAD = functional small airway disease.

## Data Availability

No new data were created for this study.
